# Beyond Old Pipes and Ailing Budgets: Systems Thinking on Twenty-First Century Water Infrastructure in Chicago

**DOI:** 10.3389/fbuil.2019.00124

**Published:** 2019-10-18

**Authors:** Laura E. Erban, Henry A. Walker

**Affiliations:** Atlantic Coastal Environmental Sciences Division, Center for Environmental Measurement and Modeling, Office of Research and Development, United States Environmental Protection Agency, Narragansett, RI, United States

**Keywords:** water infrastructure, cities, socio-environmental systems (SES), systems thinking, DSRP, reproducibility, Chicago

## Abstract

Cities are increasingly burdened by aging water infrastructure. Deferred maintenance and upgrades are compounded by emerging concerns over contaminants, extreme weather events, demographic shifts, equity, and affordability of water services. These and other evolving twenty-first century conditions prompt changes to urban water infrastructure and related systems that have wide ranging outcomes. This work demonstrates two complementary techniques for analyzing these complex systems, through the example case of Chicago. Chicago has some of the oldest urban water infrastructure in the US and supplies drinking water to more than 5 million people. Recent efforts to improve the physical and financial components of Chicago’s water system have run into a gamut of social and environmental issues. Here, a socio-environmental systems (SES) context for Chicago’s water infrastructure is structured using a rigorous systems thinking method and visual grammar to map the SES in terms of distinctions, systems, relationships and perspectives (DSRP). DSRP maps structure information about how water flows through city and how money flows through the public utilities responsible for drinking water delivery, wastewater treatment and stormwater management. Flows are evaluated, using open data and methods, over a 23-year period (1995–2017). Overall declines in water use and wastewater production are accompanied by an increase in the costs of water services, costs that support not only water infrastructure operations, maintenance and capital improvements, but also other municipal functions. Trends in the integrated data are interpreted through iterative refinement of DSRP maps to include additional components and to consider the SES from different points of view. Findings suggest that systems thinking is important for designing urban water system upgrades that are responsive to diverse socio-environmental concerns. As changes are made, transparent, reproducible methods for tracking outcomes can support analysis of differential impacts on users. The methods applied here at the city scale may be used to better understand localized, complex issues surrounding water infrastructure upgrades in Chicago and other cities.

## INTRODUCTION

Water infrastructure in the US needs significant investment. More than $1 trillion dollars is needed over the next 25 years to restore the status of the nation’s water and wastewater systems from respective D and D+ ratings (ASCE, 2017). Much of the infrastructure in place is old and was designed to address nineteenth and twentieth century problems. Evolving socio-environmental conditions of the twenty-first century—from climate change to affordability concerns—are, however, changing the design criteria and constraints (Gleick, 2000; Milly et al., 2008; Short et al., 2012; Hering et al., 2013). Investment in upgrades involves addressing complex questions: who pays, who benefits, and which redesigns will prove robust and resilient in the future?

Chicago was one of the first US cities to be plumbed, beginning in the 1850s. From that time on, Chicago has engaged in massive (in scale and expense) waterworks projects to provide fire protection, access and distribute clean drinking water, reclaim and treat wastewater (Smith, 2013). The city has been involved for more than 45 years in one of the nation’s largest public works projects to retrofit a water system: the Tunnel and Reservoir Project (TARP). TARP was initiated in 1972 to deal with frequent flooding and combined sewer overflows. It is projected to continue until 2029, at an estimated total cost of $3.8 billion (MWRD, 2017).

In the meantime, new issues and responses have come to the fore. The upper Midwest is getting more precipitation during more intense storms (Angel and Huff, 1997; Easterling et al., 2017). Federal spending on water infrastructure has dwindled, pushing more costs onto local governments (CBO, 2015). Local budgets are increasingly burdened by other commitments. Chicago’s municipal pension plan was, as of 2016, on a path which “guarantees insolvency ... in the near future” (MEABF, 2016), a potential crisis that is being partly mitigated using the city’s water revenues. New ordinances around on-site stormwater management and investments in decentralized green infrastructure have provided support to centralized (or gray) water infrastructure, along with co-benefits (see, e.g., CoC, 2014).

Investment in urban water infrastructure thus occurs in the context of a complex and dynamic socio-environmental system (SES). Diverse factors, operating at a range of scales, influence the delivery of water services and outcomes for users. This work presents a novel application of two complementary methods for analysis of system configuration and performance. It (1) tracks flows of water through Chicago alongside countercurrent flows of money through the utilities responsible for its drinking, wastewater and stormwater infrastructure, over the past two decades, using open data and methods and (2) interprets trends in these data by developing structured information maps of the SES from which they emerge. Far beyond the scope and scale addressed here, these methods can be used to better understand a wide range of issues that manifest as a city endeavors to update its water infrastructure.

### A Short History of Chicago’s Plumbing

The city of Chicago was incorporated in 1837 on top of a wet prairie and glacial lakebed. It faced drainage problems from the beginning. The streets were raised between 4 and 14 feet (Cronon, 1991) in order to shed stormwater and lay sewer pipes that could drain to the Chicago River, and ultimately to Lake Michigan. Sewage soon fouled the Lake, which was and remains Chicago’s drinking water supply. Repeated epidemics of waterborne disease led the city to move its drinking water intake to a crib 2 miles offshore, connected by a tunnel under the Lake that was completed in 1867.

Chicago’s rapid early growth soon outpaced the capacity of its water infrastructure to maintain sanitary conditions. In 1889, the Sanitary District of Chicago was created and charged with building the Chicago Sanitary and Ship Canal (CSSC) to divert sewage away from the Lake. The CSSC opened in 1900, reversing the flow of the Chicago River (now toward the Mississippi River) and using Lake Michigan to flush the city. But the CSSC and subsequent canals could not keep up with Chicago’s waste, and by the 1920s, construction of wastewater treatment facilities was underway. The need for sewage treatment capacity was exacerbated in 1930 by a Supreme Court decree limiting Illinois’ withdrawals from Lake Michigan.

Despite the incredible scale of Chicago’s efforts to manage wastewater and stormwater, early problems have persisted. By the 1940s, Chicago’s combined sewer system, which many older cities also have, was regularly overflowing (Moser, 2013). To contain untreated wastewater, the TARP project was initiated in 1972. TARP currently captures about 85% of would-be combined sewer overflow (CSO; MWRD, 2017) using 109.4 miles of deep tunnels, two reservoirs and part of a third constructed to date (nearly 11 billion gallons of storage), but even a modest rainstorm can still trigger CSO events (Hawthorne, 2011; CoC, 2014). Flooding, above ground and in basements, remains a chronic issue that is uncorrelated with floodplains (CNT, 2014).

### Modern Perspectives on Chicago’s Water System

In early 2012, Chicago Mayor Rahm Emanuel promoted a $1.4 billion investment in water and sewer infrastructure saying, “We know that, as long as our city rests on a twentieth century foundation, we won’t be able to compete in a twenty first century economy” (Ivanova, 2012). As part of this investment, the city’s Department of Water Management (DWM), the utility responsible for drinking water distribution and sewer collection services, received funding to replace aging water mains at a rate equivalent to when they were installed over 100 years ago (DWM, 2012). Some residents became concerned that water main replacements had caused elevated lead in their drinking water and filed a class action lawsuit (Berry v. City of Chicago. 2016 CH 02292, 2018). Though the suit was recently dismissed, areas of physical disturbance in the distribution system have been associated with elevated lead levels in Chicago homes (Del Toral et al., 2013).

At the same time, spending on infrastructure upgrades triggered significant increases in the costs of water and sewer services. Although Chicago pays considerably less for water than many other US cities (CoB, 2018), large multi-year rate hikes associated with the 2012 capital improvement program had severe impacts for some low-income residents in Chicago and in suburbs that buy water from the city (Gregory et al., 2017). Some towns, facing budgetary shortfalls, have not paid for city water in years and are being sued (Koeske, 2018). Confronting mounting liabilities of their own, the Mayor of Chicago and City Council approved a new and controversial tax in 2016. The tax was added to water and sewer rates in order to stabilize municipal pensions on the brink of insolvency, while the city ramps up its pension contributions using “dedicated revenue streams” (CoC, 2018).

### Systems Thinking on Urban Water Infrastructure

Recent efforts to replace old pipes and shore up ailing budgets in Chicago (see section Modern perspectives on Chicago’s water system) highlight interdependencies with a broader SES. From a systems perspective, water services in the city are delivered jointly by physical and social infrastructure. People work for institutions, develop laws and regulations, raise and spend funds, vote for leaders, and engage in many other activities that influence water use and management at household to community and larger scales. These activities are also subject to biophysical constraints. The environmental setting and the urban development history (see section A short history of Chicago’s plumbing) fundamentally impact water availability and quality, as well as the needs around and capacity for water distribution and drainage in Chicago, as in any city.

Synthesis of such disparate influences has adopted a range of forms. There is no consensus on how to model the complexity of urban water systems, but rather a variety of tools, developed from different disciplinary perspectives. Some account for mass conservative quantities in terms of stocks and flows, while others examine qualitative measures. Case studies make use of mixed methods, including stakeholder interviews, data-driven narratives and primary analysis, to derive insights into urban water governance, decision-making, and management transitions (e.g., Hughes et al., 2013; Hornberger et al., 2015; Treuer et al., 2017). Life-cycle assessments perform comprehensive accounting of urban water infrastructure technologies, in terms of physical components and materials consumption, motivated by socio-environmental implications (e.g., Lundie et al., 2004; Loubet et al., 2014). Simulation models of urban water systems represent social and biophysical processes with coupled equations, integrating complex dynamics to varying degrees (see Bach et al., 2014 for a recent review). Each approach is suited to specific applications and involves inherent tradeoffs in terms of scope, resolution, time commitment, and type of expertise.

Despite progress in more holistic study of urban water systems, interdisciplinary, interagency practice to improve them remains elusive (e.g., Hering et al., 2013; Mukheibir et al., 2014; Dunn et al., 2017). Enhanced cross-sectoral collaboration is needed to accomplish favorable triple bottom line (social, environmental, and economic) outcomes as these systems are redesigned. Impediments to more synergistic urban water management include limits to institutional authority and capacity, and a breadth of specialized roles in this space—from engineering to finance and public administration—factors that are compounded by “a lack of trained systems thinkers” (Mukheibir et al., 2014). Systems thinking is itself a sprawling field, defined differently by many scholars, encompassing a wide range of methods and tools (see Monat and Gannon, 2015). Practically speaking, however, systems thinking offers “an antidote to silos” (Williams et al., 2017). The persistence of silos stems in part from confusion over how to *do* systems thinking.

One straightforward approach, based in cognitive science, recognizes thinking as a complex adaptive system acting out four simple rules: making Distinctions, organizing Systems, recognizing Relationships, and taking Perspectives (DSRP; Cabrera et al., 2008; Cabrera and Cabrera, 2015). DSRP involves diagramming, or mapping, a given system according to these rules, with a consistent visual grammar. Maps explicate thinking, structure information, organize space for refinements, and represent a given system from multiple perspectives. They expose mental models of how a system works and present opportunities to identify missing components or feedbacks. The exposed model may then serve as a framework for further analysis with other tools. This rigorous systems thinking method was recently used to understand critical failures in the Flint water crisis, when lead leaching from pipes followed a financially motivated decision to switch the Michigan city’s water supply (Sokolow, 2017).

This work builds upon the Flint case and other studies (Vachliotis et al., 2014; León and Calvo-Amodio, 2017; Piggot-Irvine et al., 2017; Jagustović et al., 2019), by demonstrating conjunctive use of DSRP and open data synthesis to better understand urban water infrastructure issues in a broader context. It takes the perspective that the acute problems that Chicago, and other cities, seek to address—lead in drinking water, CSO events, or adverse effects of rate increases, for example—are emergent properties of a complex SES. A systems perspective can provide context for making decisions that affect a city’s water infrastructure and related systems, as well as for anticipating outcomes. Evaluation of actual outcomes depends on how data is synthesized. Here, context and data are analyzed in tandem, to structure thinking about the workings of major city’s water system and interpret indicators of its physical and financial performance.

## METHODS AND RESULTS

Prior work explored the current limitations to comprehensive and open accounting of water flow through Chicago’s sevencounty regional planning area (Erban et al., 2018). The R package *CityWaterBalance* (Erban, 2017) was developed to acquire data from federal web services, merge local sources, and estimate unmeasured flows using a mass-conservative model, applied to water years 2001–2010. Analysis of financial flows support water services was beyond the scope of that effort, and the same approach was not applicable: money does not observe the law of conservation, accounting data is less accessible, and reporting standards change over time. This work used *CityWaterBalance* and the data sources given in Table 1 to analyze water flows in Chicago and Cook County through 2017. Concurrent financial data were manually extracted from the utilities’ Comprehensive Annual Financial Reports (CAFR). A computational R notebook (see the Supporting Materials) was used to document the process in plain language and machine-readable code, integrate the data (also provided) and generate time series plots (see Figures 3, 4).

**FIGURE 1 |:**
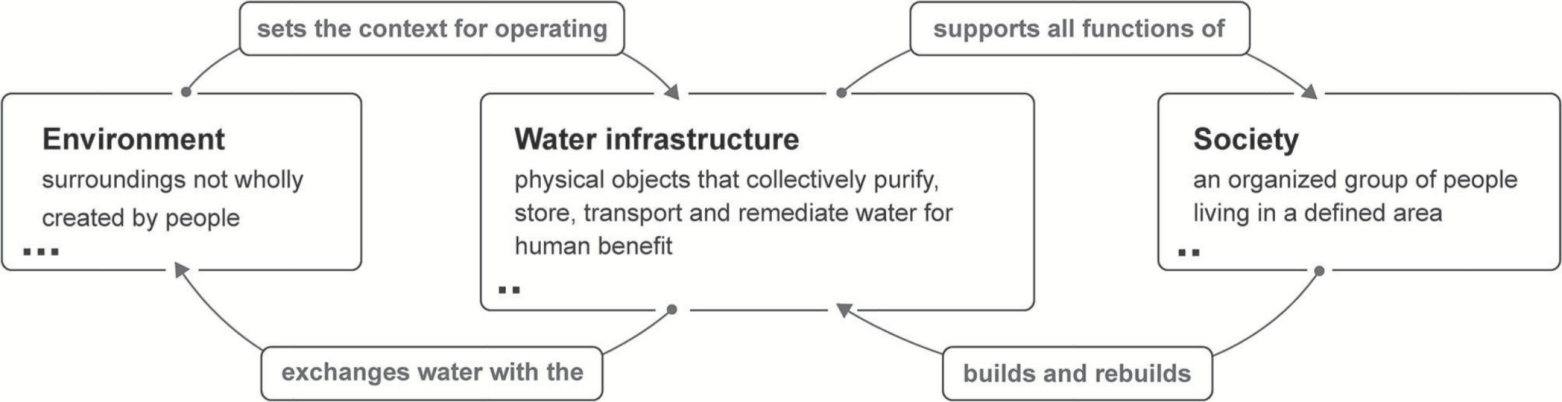
DSRP map of urban water infrastructure in an SES context. Dots indicate the number of distinctions made for system parts. Parts are expanded in subsequent maps. Map originated with Plectica.

**FIGURE 2 |:**
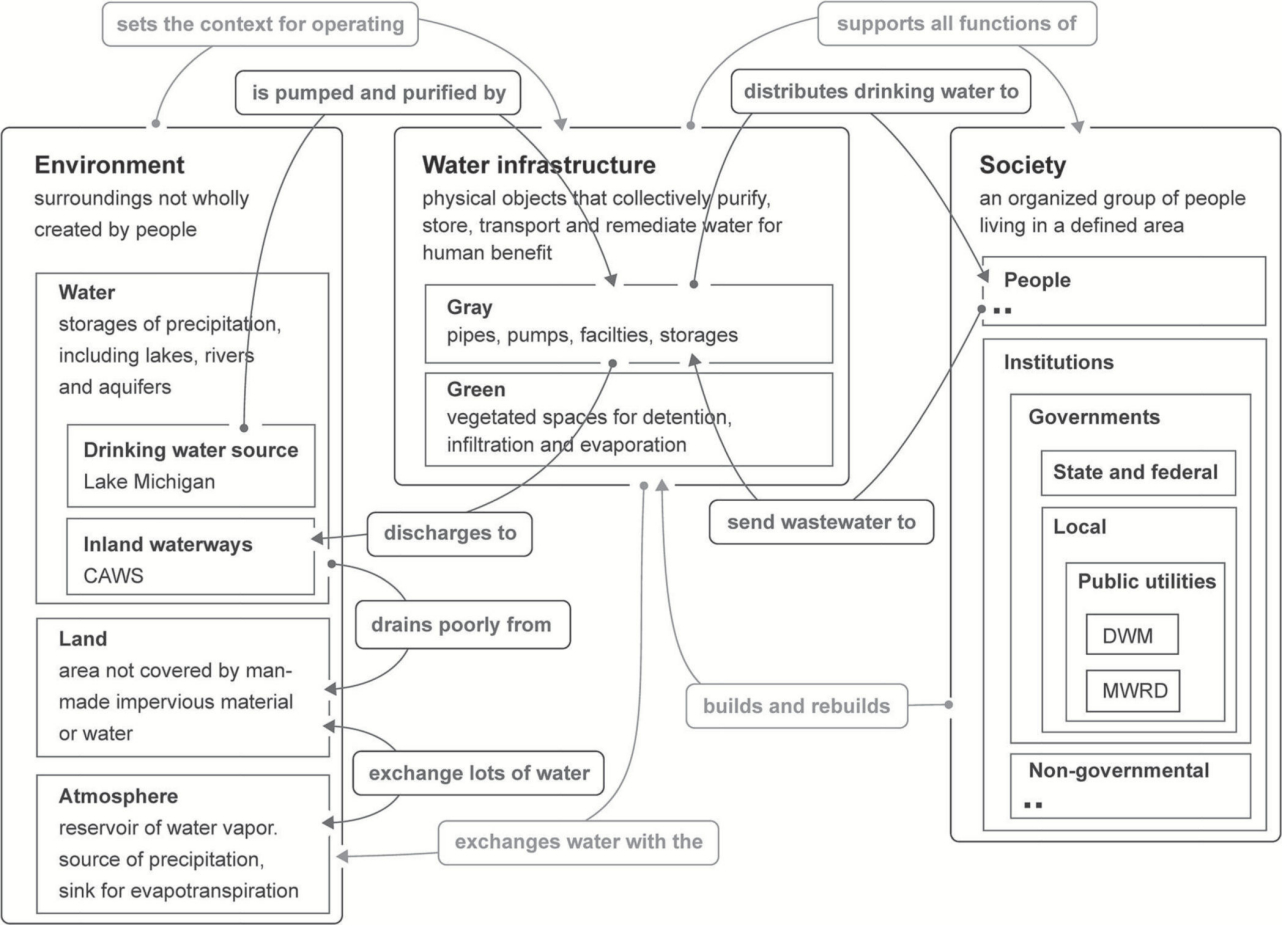
Refinement of the general SES map (Figure 1) for the Chicago case, focusing on flows of water. High-level relationships from Figure 1 have been lightened to put visual emphasis on new distinctions and relationships. Dots in a system box indicate parts that are expanded in other figures. Map originated with Plectica.

The system maps (Figures 1, 2, 5) were generated using Plectica, an interactive, online mapping platform based on DSRP’s visual grammar (see section Systems thinking on urban water infrastructure). The first DSRP map (Figure 1) lays out a high level SES context for urban water infrastructure. It contains three primary distinctions: Environment, Water Infrastructure, and Society. Each of these distinctions is also system made up of parts. The number of parts that have been distinguished is indicated by dots in the lower left corner of the system boxes. Parts are collapsed here and expanded in subsequent maps. Relationships between the three systems are distinguished with labeled arrows. Although distinctions may appear to be fixed in these maps, they are in fact dynamic: systems, relationships and perspectives (DSRP) change with time. Perspectives have not been explicitly distinguished in the map, though one is implied: that of the authors.

The initial SES map was further developed (Figure 2) to refine an understanding of how water moves through Chicago. There are two public utilities with immediate responsibility for water delivery and reclamation. The City of Chicago’s Department of Water Management (DWM) supplies the city and 125 suburban municipalities with nearly 1 billion gallons of drinking water daily. The water is taken from Lake Michigan, purified at two filtration plants, and distributed to more than 5 million people. DWM is responsible for a network of nearly 9,000 miles of water and sewer mains, the latter of which connect to intercepting sewers owned and maintained by the Metropolitan Water Reclamation District of Greater Chicago (MWRD, formerly the Sanitary District).

MWRD, a special-purpose district, reclaims and treats wastewater in the majority of Cook County (93%; MWRD, 2016). Chicago makes up approximately one quarter of Cook’s area and half of the county’s residents. MWRD operates seven wastewater treatment plants and manages the TARP system. It also physically regulates water levels and quality in the Chicago Area Waterways System (CAWS), some 90-plus miles of interconnected natural and manmade channels that ultimately convey wastewater effluent and stormwater away from the city (Duncker and Johnson, 2015). MWRD was delegated authority over stormwater in all of Cook County by the Illinois General Assembly in 2004 and is a major developer of green infrastructure.

**FIGURE 3 |:**
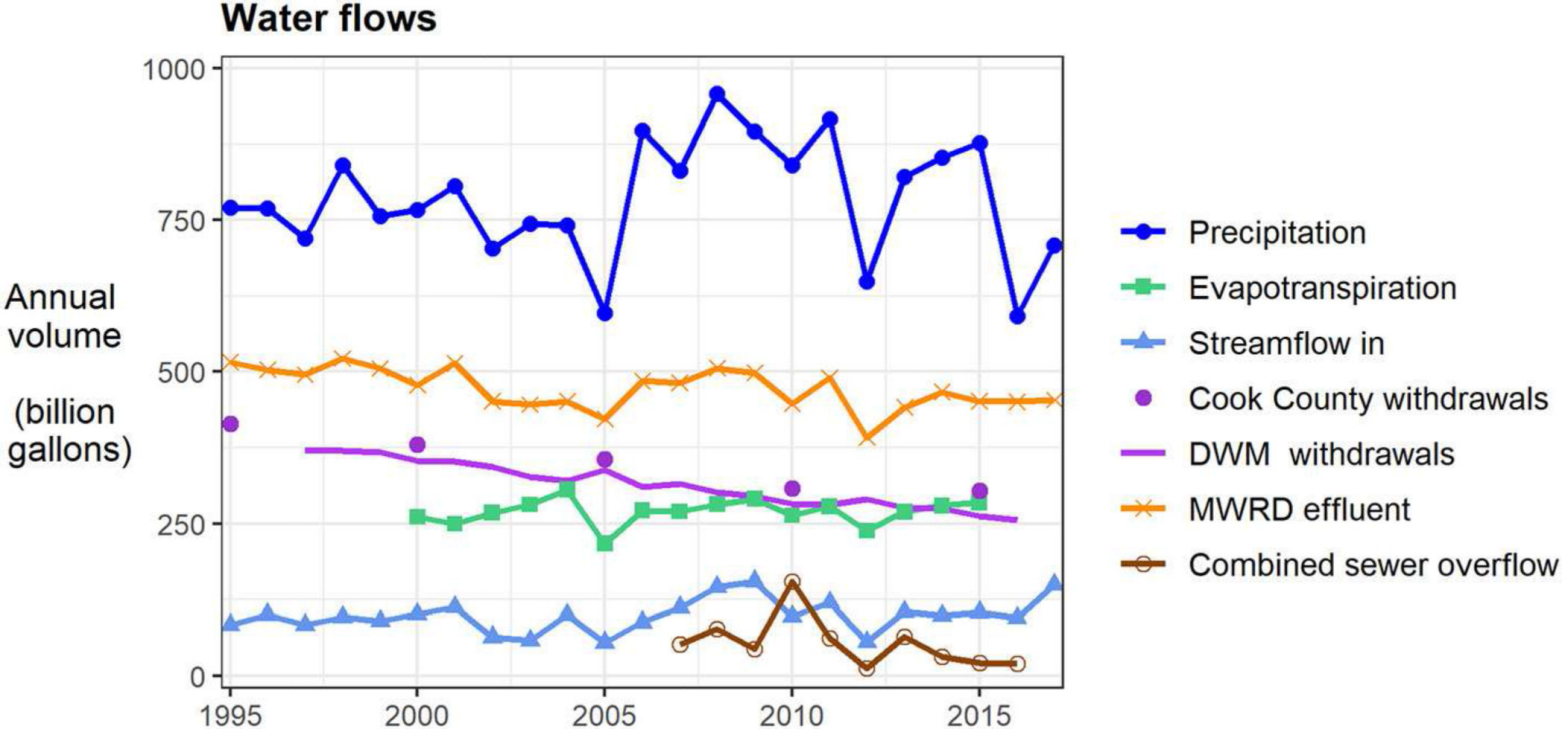
Recent trends in major environmental and manmade flows of water. All data are continuous annual totals, except for Cook County withdrawals (data is given at 5-year intervals). The MWRD effluent time series shows a superposition of trends in precipitation (short-term variability) and DWM withdrawals (long-term decline). Annual CSO totals reflect cumulative conveyance and treatment capacity during individual wet weather events, during which conditions vary considerably. They are not expected to precisely track annual precipitation. Still, CSO and precipitation minima co-occur in 2012. Additional information on flows of water in greater Chicago can be found in Erban et al. (2018). Data sources for this figure are reported in Table 1.

Surrounding this built infrastructure is an environment that determines how water moves through the region at a larger scale. The atmosphere delivers precipitation, which is the largest source of water flowing into Cook County (Figure 3). Drainage of this water overland is limited by low topographic relief and low channel density (Duncker and Johnson, 2015). Infiltration belowground is limited by widespread impervious surfaces, low permeability soils, and high water tables (Morrow and Sharpe, 2009). Much of the water that infiltrates is returned to the atmosphere through evapotranspiration (Jones, 1966; Yeh and Famiglietti, 2009) and streamflow provides another major outlet (Duncker and Johnson, 2015; Erban et al., 2018). Imbalances in water delivered and removed by the environment creates stormwater, flooding and drought, among other conditions that are dealt with by Chicago’s water infrastructure and its people.

People impose other variable demands on water infrastructure. Withdrawals for public supply in Cook County have been declining for decades (Figure 3). Cook’s withdrawals are primarily made up of water pumped from Lake Michigan by DWM, which is the second largest inflow to the county. DWM withdrawals, which rise slightly in dry years (e.g., 2005, 2012), have fallen overall due to a convolution of factors in two broad categories: (1) leak reduction in the distribution system by the utility and (2) declining user demand (DWM, 2008, 2012). MWRD effluent (i.e., treated wastewater) has also declined, though to a lesser extent during the recent (post 2006) wet period, likely due to an increase in stormwater in the combined sewer system. Additional stormwater appears to be largely accommodated by TARP, as combined sewer overflows have fallen, overall.

**FIGURE 4 |:**
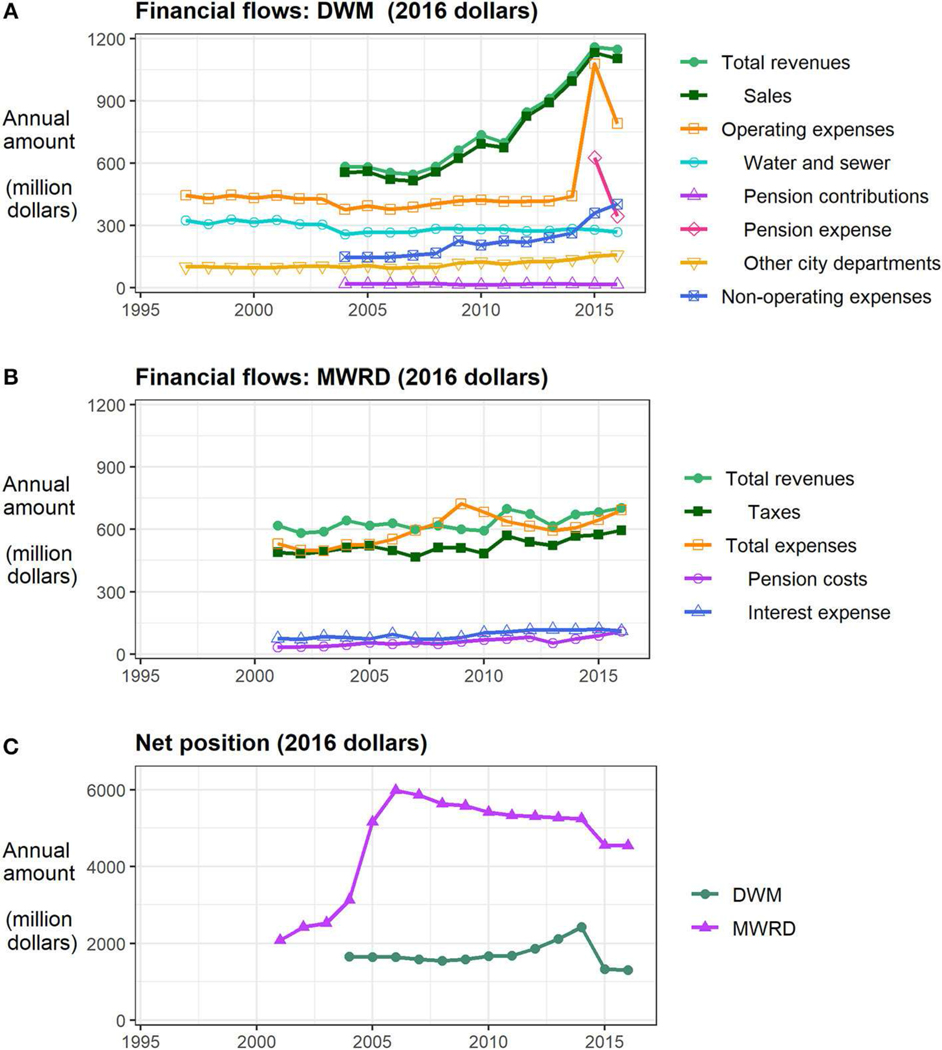
Recent trends in flows of money through Chicago’s drinking and wastewater utilities **(A,B)**, along with changes in net position **(C)**. All data are annual totals, adjusted for inflation (2016 dollars). Data sources are reported in Table 1.

People also control how water infrastructure is supported financially (Figure 4). Funds flow to DWM and MWRD for two general purposes: operations and capital improvements. DWM revenues derive primarily (>95%) from water and sewer bills, supplemented by fees for services and other sources. Revenues flow to DWM’s Water and Sewer Funds (or “Enterprise Funds,” from the City’s perspective) to support DWM and other city departments. MWRD derives most of its revenues (>80%, MWRD, 2016) from property taxes and the remainder from a mix of sources (fees, land rentals, grants, interest, other). Capital improvements for both agencies are largely funded through debt, in the form of bonds and state revolving funds (SRF). Changes in revenues, expenses and debt are documented in CAFRs, which articulate a multitude of perspectives on the status of the funds managed by each agency, in terms (and sometimes values) that can be inconsistent within and between lengthy biennial reports.

Accounting and reporting differences obscure a direct comparison of the two utilities’ finances. However, trends can be discerned in the aggregated flows of money through them (Figures 4A,B). To keep language consistent with the CAFR reports, some terms vary across subplots. All trends presently discussed are adjusted for inflation (2016 dollars). DWM’s revenues have nearly doubled since 2004, due to rate increases for water and sewer nearly every year since 2008 (averaging ∼12% per year). Expenses have also grown. Operating expenses spiked in 2015 due to a new pension expense (discussed in more detail below). Non-operating expenses, including debt service, rose considerably, if more gradually, over the reporting period. Other city departments have received an increasing amount of DWM’s revenues. Spending has fallen on core water and sewer-related operations (calculated as operating expenses less the other three sub-categories). By comparison, MWRD’s revenues and expenses have risen more gradually during the period. There is a distinct temporary rise in total expenses beginning in 2009 due to maintenance costs (MWRD, 2010). MWRD’s pension cost and interest expense on outstanding bonds have also risen.

The overall financial well-being of each agency is indicated by its net position (Figure 4C), or the reported difference between total assets and liabilities. The net position of each agency increased significantly with major capital improvements (2005–2006 for MWRD, 2012–2014 for DWM) and declined sharply in 2015 with the implementation of new accounting standards that require CAFRs to recognize long-term pension obligations as a liability (GASB 68; see GASB, 2012). Accounting for this liability makes balance sheets more accurate (and transparent) but does not change actual pension contributions. DWM’s pension contributions have remained fairly steady and are considerably lower than the estimated pension expense (see Figure 4A), or the amount needed to fully fund this obligation. MWRD does not report pension expense as a line item, but its reported “pension cost” has nearly doubled during the reporting period (Figure 4B).

**FIGURE 5 |:**
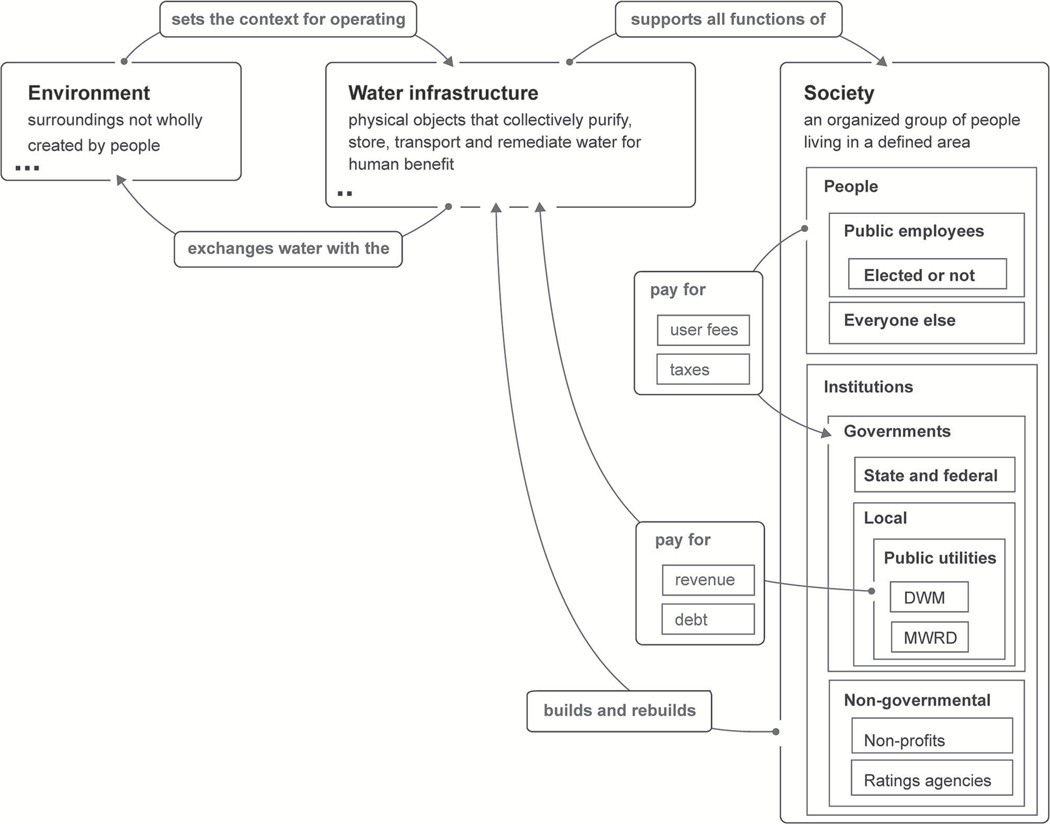
Refinement of the SES around water infrastructure in Chicago, focusing on financial relationships. Dots in a system box indicate parts that are expanded in other figures. Map originated with Plectica.

In the process of parsing financial flows, a second refinement of the initial SES map was developed (Figure 5). This financial perspective on the SES illustrates a disconnect between what people and utilities “pay for.” People pay for water services through user fees and taxes, but the flow paths for these funds are indirect. Rather than paying providers directly for services rendered, funds are routed through public agencies that use them to deliver water and other services. The disconnect further complicates, and obfuscates, the workings of this complex system. The entanglement of Chicago’s Enterprise and general accounts, and lapses in payment for water from suburbs are symptoms of this structural problem. Each issue could be mapped in more detail by the parties involved in the transactions for improved understanding or potential resolution.

Perspectives of people in this system, though not depicted in the maps shown here, are also essential to how it works. As rate-payers and tax-payers, people influence water infrastructure operations and municipal revenue generation. They adjust how they use water and even where they live based in part on feedback from these costs. They also influence higher level controls over how revenues are raised and spent from another widely shared point of view: as voters. Elected officials (a subset of residents with a different point of view) set rates, propose bond measures, and distribute financial resources among competing needs. Critical competing needs include unfunded pension liabilities, particularly from the perspective of public workers (another subset). The status of pension funds in turn impacts bond ratings (see, e.g., Chapman et al., 2017 for a ratings agency perspective), which determine the cost of water infrastructure-related debt for current and future residents.

## DISCUSSION

Urban water infrastructure is part of a dynamic SES involving many other systems within and beyond a city. In Chicago, drinking water distribution and sewage collection systems are maintained by a department of the city government (DWM), which routes wastewater to another utility (MWRD) serving the larger county. As demand for water has fallen over the past two decades, DWM provides fewer gallons at increased cost. Rising costs are not unexpected, based on lost sales and the city’s efforts to replace old pipes. But DWM’s revenues, which are mostly water and sewer fees, fund more than just maintenance and improvement of Chicago’s water infrastructure: they supplement other city departments and pension deficits. Pension liabilities have only recently been estimated and disclosed (in 2015), and not yet fully funded, leaving open the possibility of future escalation in the cost of water services to pay for other municipal obligations. Many local government entities share a tax base with MWRD. The overlap is a potential vulnerability for MWRD’s revenues that affects its financial outlook, from the perspective of bond ratings agencies. Bond ratings influence the cost of borrowing. In addition to conditions that are locally controlled, ratings increasingly account for climate change risks (Mathiesen, 2018).

Following the water and money through Chicago’s water utilities in this way, with structured information maps coupled to quantitative assessment, yields a tractable, city scale perspective on a major urban water system. Many critical system components have been explicated, and functions evaluated, while many others are important from other perspectives or at other scales. Daily decisions by utility staff affect the delivery of centralized water services. Land use change influences water flows locally. Professional societies set standards for water infrastructure that guide what is built and how it is maintained. Episodic decisions by lawmakers and regulators make tweaks to other system components. Non-governmental organizations lobby for and against these tweaks. Each change can feel significant, depending on one’s perspective, but the SES perspective underscores considerable inertia. The environmental context, the physical structures that allowed the city to grow (becoming more complex) and on which its current residents depend, and the social system that pays for water services are well-developed and resistant to change by individual agents or agencies (Forrester, 1969; Kiparsky et al., 2013; Mukheibir et al., 2014; Ma et al., 2015).

Coordination of efforts to improve not just water infrastructure, but also related socio-environmental conditions, can benefit from systems thought and practice. While many methods exist, one simple approach uses four cognitive rules to map systems: drawing Distinctions, organizing Systems, recognizing Relationships and taking Perspectives (DSRP; Cabrera et al., 2008; Cabrera and Cabrera, 2015). DSRP maps can coax an otherwise dizzying and divergent set of ideas into concise, explicit representations. This general method is applicable to any subject or scale and is readily understood by any user. As such, it has potential to enhance collaboration by supporting more integrative, cross-sectoral understanding of multi-faceted issues. In addition to a number of such issues already discussed, Chicago’s regional planning agency has stated concerns over how to maintain water infrastructure in depopulating neighborhoods (CMAP, 2017). Maps of this issue developed by groups of stakeholders, and from a range of perspectives, would structure a richer understanding of the problem than is held by any particular agency or individual. A mapping process that engages local partners could elicit neighborhood-specific solutions.

As solutions for water and related critical infrastructure are implemented, cities increasingly seek to track and assess outcomes. Data are increasingly exposed through open data portals, from local to federal, or the websites of individual agencies. For cities throughout the US, the kinds of data used here are generally available, along with a wealth of other metrics. However, urban analytics stands to benefit not only from better data availability, but accessibility and informative synthesis. The basic time series shown here are not straightforward to produce. Aggregating the data is an exercise itself in systems thinking. Data reflect perspectives (e.g., what to report on a financial balance sheet), part-whole systems (e.g., categories of water withdrawals and use), and relationships can be obscured (e.g., account transfers). Data alone do not generate understanding; additional, structured information about the system that produces them is needed to make sense of values and trends. Where data is lacking due to resource constraints, systems thinking can aid in determining what to collect and who will benefit. This work has considered only a subset data relevant to understanding recent changes in Chicago’s water system and outcomes for its people. But it has demonstrated a way to approach systemic assessment, that can be applied to any set of indicators, as a city works toward equitable and adaptive redesigns.

## CONCLUSIONS

Infrastructure investment is a continuous process embedded in a complex, evolving SES. Upgrades to centralized water infrastructure can solve problems for many people in the near-term, but complex systems quickly compensate for change. Despite herculean efforts to modernize Chicago’s water system throughout much of the nineteenth and twentieth centuries, the city soon outgrew each solution. Today Chicago, like many US cities, finds itself maintaining a legacy water system while dealing with emerging concerns that vary among current residents according to their location and socioeconomic status.

This work has begun to map the SES involved in water delivery, reclamation and treatment in Chicago using a systems thinking method based on simple rules (DSRP). DSRP renders a high-level understanding of this complex system tractable and promotes communication of how it works. Although here it was applied citywide, DSRP is independent of scale, and may be used to understand components of Chicago’s water system in greater detail. The analysis provides a concise and coherent understanding of how water services are delivered to residents amidst competing municipal demands. Actions taken from diverse perspectives, within and beyond the city, exert bottom-up and top-down control over changes to its water infrastructure, with differential outcomes for users.

The process of tracking outcomes can be improved by more transparent accounting. Water can be tracked with relatively simple accounting principles, but financial accounting changes with actuarial and reporting standards, creation of revenue streams and accounts. Although this study used publicly available information, it could be far more accessible. Greater transparency— brought about by governmental accounting standards, open data repositories and computationally reproducible research methods—can shed light on otherwise obscure and multi-faceted dynamics. Systemic assessment of municipal data may reveal opportunities for water infrastructure redesigns that are responsive to wide-ranging social and environmental concerns of the twenty-first century and beyond.

## Supplementary Material

Sup1

## Figures and Tables

**TABLE 1 |:** Data sources for Figures 3,4*.

Variable	Availability	Source

Precipitation*	Online, API	PRISM, monthly mean of gridded product within Cook County; available from USGS Geo Data Portal
Evapotranspiration*	Online, API	SSEBop, monthly mean of gridded product within Cook County; available from USGS Geo Data Portal
Streamflow in*	Online, API	USGS NWIS, daily mean streamflow at selected gauges
Cook County withdrawals*	Online, API	USGS NWIS, annual water use by county, 5-year reporting
DWM withdrawals	Online, pdf	Annual pumpage from Water Fund CAFRs for 2016, 2006. Available at: https://www.cityofchicago.org/
MWRD effluent	Online, xls	Daily effluent for each water reclamation plant Available at: https://www.mwrd.org/
Combined sewer overflow	Offline, xls	CSO events by MWRD intercepting area Available upon request
DWM revenues, expenses, and net position	Online, pdf	Annual data from Water and Sewer Fund CAFRs for 2016, 2006. Available at: https://www.cityofchicago.org/
MWRD revenues, expenses, and net position	Online, pdf	Annual data from MWRD CAFRs for 2004–2016, every 2 years. Available at: https://www.mwrd.org/

* retrieved using R package CityWaterBalance (Erban, 2017).
