# Supplementary material for: Beyond Old Pipes and Ailing Budgets: Systems Thinking on Twenty-First Century Water Infrastructure in Chicago
Source: Front Built Environ. Author manuscript; Available in PMC 2021 Mar 18. (PMC7970536; doi:10.3389/fbuil.2019.00124)
Supplement: Sup1 [file NIHMS1541468-supplement-Sup1.zip › Supplement/Supplement_SES_water_Chicago.nb.html]

Code 

- Show All Code
- Hide All Code
- Download Rmd


```
library(CityWaterBalance)
library(dataRetrieval)
library(leaflet)
library(ggplot2)
library(tidyverse)
library(xts)
library(gridExtra)
library(knitr)
```


### Supplementary material for “*Beyond old pipes and ailing budgets: systems thinking on 21st century water infrastructure in Chicago*”

#### Authors: Laura E Erban and Henry A Walker

This notebook provides the steps used to gather, summarize and plot the water and financial data shown in the paper (Figs 3-4). With the supplementary data files and the code given below, one can reproduce our work.

#### 1. Water

##### 1.1 Environmental flows of water

The code chunk that follows was used to acquire monthly estimates of precipitation (prcp) and evapotranspiration (et) rates from gridded model output (models are PRISM, SSEBop, respectively). Model output was retrieved from the USGS’s Geo Data Portal through the end of 2015 and spatially averaged within the boundaries of Cook County (county boundaries are dashed lines in the map below, which is centered on Cook). We calculated annual totals (as depth of water) through 2015 and manually added precipitation totals for 2016 and 2017 using data for O’Hare airport from weather.gov. We converted depths to volumes by multiplying by the land area of Cook County (i.e., excluding Cook’s area in Lake Michigan). We retrieved daily streamflow from USGS’s NWIS for streamgauges near Cook’s boundaries (see map). Streamflow records are not ideally located, and contain gaps. The code specifies how we accounted for those issues and summarized total annual streamflow into Cook County.


```
# get precipitation (prcp)
prcp <- getPrecipitation('1995-01-01', '2015-12-01', 'sample:Counties', 'FIPS','17031')
names(prcp) <- c('date', 'rate', 'var', 'stat', 'units')
psum <- prcp %>% 
  mutate(year = lubridate::year(date)) %>%
  group_by(year) %>%
  summarise(ptot = sum(rate))

# add data from weather.gov for O'Hare 
psum <- rbind(psum, c(2016, 35.97*25.4), c(2017, 43.1*25.4))

# get evapotranspiration (et) 
et <- getEvapotranspiration('1995-01-01', '2015-12-01', 'sample:Counties', 'FIPS','17031') 
names(et) <- c('date', 'rate', 'var', 'stat', 'units')
esum <- et %>% 
  mutate(year = lubridate::year(date)) %>%
  group_by(year) %>%
  summarise(etot = sum(rate))

atm <- left_join(psum, esum)

# convert prcp, et rates to volumes
area <- 2450     # land area of cook county (km2)
cf <- 0.003785   # cubic km per billion gallons  
atmvol <- atm %>%
  mutate(ptot = (ptot/1e6)*area/cf) %>%
  mutate(etot = (etot/1e6)*area/cf)

# get streamflow and estimate values in data gaps
streamgages <- c('05528100','05529000','05536195','05535070', '05534500', '05535500')
s <- getStreamflow('1995-01-01', '2017-12-31', streamgages)
s2 <- gapfillStreamflow(s, list(c('05529000','05528100'), c('05536195','05529000'), c('05535070','05536195'), c('05534500','05536195'), c('05535500','05536195')))
sf <- apply.yearly((s2$flows * 60*60*24), FUN = colSums)   # convert to cubic ft / day and sumarize by year
sf <- sf * 7.48052 / 1e9                                   # convert streamflow to billion gall / year

# deal with streamgage location issues. first two gages are on des plaines, ~ equidistant on either side of Cook County border.  Upstream avgs 70% of downstream.  Applying 85% to downstream gage (complete record) to split the difference
sest <- data.frame(lubridate::year(index(sf)), as.vector(sf[,2]*0.85+sf[,3]+sf[,4]+sf[,5]+sf[,6]))
names(sest) <- c('year', 'streamflow_in')

env <- left_join(sest, atmvol)
```

##### 1.1.2 Map showing county boundaries, rivers and streamgage locations


```
streamgages <- c('05528100','05529000','05536195','05535070', '05534500', '05535500')
sg <- readNWISsite(streamgages)

# lat lons for streamgauges
sglocs <- tibble(lat = sg$dec_lat_va, lon = sg$dec_long_va, site = sg$station_nm)

leaflet(sglocs) %>% 
  addProviderTiles("Esri.WorldTopoMap") %>%
  addMarkers(~lon, ~lat, popup = ~as.character(site), label = ~as.character(site)) %>%
  addScaleBar()
```

##### 1.2 Manmade flows

The code chunk below loads the data gathered above and additional data on manmade flows of water, most sources of which are not federated. Water use data at the county level are published by USGS at 5-year intervals from 1985-2015. Code below shows how to retrieve these data. Wastewater effluent (i.e. treated wastewater) data was manually downloaded from MWRD’s Water Reclamation Plant Data, available as Excel spreadsheets by day and plant going back to 1982. Combined sewer overflow (CSO) volumes were obtained through a written request to MWRD. Pumpage by DWM from Lake Michigan is disclosed in CAFRs (discussed more in the financials section below). We aggregated these data outside of this workbook and prove annual summaries, along with the environmental flow data from above, in the supplementary file “Water\_Data.csv”.


```
# ------- retrieve public supply withdrawals -------
wu <- getWaterUse(c('IL'), list(c('Cook')))

# aggregate surface, groundwater.  covert to BG / yr
pubw <- (wu$swf$Public + wu$gwf$Public) * 365/1000
pubw <- data.frame(wu$swf$year, pubw)
names(pubw) <- c('year', 'public_supply_BG')
# --------------------------------------------------

# load all water data
water <- read.csv("Water_Data.csv")
```

##### 1.3 Water flow data plotted (manuscript Fig. 3)


```
# load all water data
water <- read.csv("Water_Data.csv")

# 1. Plot bulk water flows
m <- gather(water, var, value, -year)
m$var <- factor(m$var, levels = unique(m$var))

shapes = c(16, 15, 17, 19, NA, 4, 1)

p <- ggplot(m, aes(x = year, y = value, group = var)) +
  geom_line(aes(colour = var), size = 1) +
  geom_point(aes(colour = var, shape = var), size = 2) +
  scale_shape_manual(values = shapes, guide = 'none') +
  scale_x_continuous(limits = c(1995, 2017), breaks = seq(1990, 2017, 5), expand = c(0.01, 0.01)) +
  scale_y_continuous(limits = c(0, 1000), expand = c(0.02, 0.02)) + 
  theme_bw() +
  scale_colour_manual(name = '', values = c('blue', 'seagreen3', 'cornflowerblue', 'darkorchid', 'darkorchid2', 'darkorange', 'darkorange4'), labels = c('Precipitation', 'Evapotranspiration', 'Streamflow in', 'Cook County withdrawals', 'DWM  withdrawals', 'MWRD effluent', 'Combined sewer overflow')) +
  ggtitle(paste("Water flows")) +
  labs(x = "", y = "Annual \n volume \n \n (billion \n gallons)") +
  guides(colour = guide_legend(override.aes = list(linetype = c(1, 1, 1, 0, 1, 1, 1), shape = shapes))) +
  theme(plot.title = element_text(size = 12, face = "bold"),  legend.text = element_text(size = 10), legend.position = "right", axis.title.y = element_text(angle = 0, vjust = 0.5))

plot(p)
```

#### 2. Money

Flows of money through the two water utilities serving Chicago (DWM, MWRD) were extracted from Comprehensive Annual Financial Reports (CAFR). Financial flows through DWM were summed from values in two sets of biennial CAFRs for Chicago’s Water and Sewer Funds, published online for 2006-2016. Values were extracted from a single set of MWRD CAFRs, available on a biennial basis from 2004-2016. This was also a manual process, results of which are provided in “Money\_Data\_DWM.csv” and “Money\_Data\_MWRD.csv”. In the code chunk below, they are corrected for inflation (relative to 2016), using the Consumer Price Index for All Urban Consumers (CPI-U) Chicago-Naperville-Elgin available from the US Bureau of Labor Statistics.

##### 2.1 Financial history - DWM


```
tiff("figure3.tiff", width = 7, height = 3.5, units = 'in', res = 300, compression = 'lzw')
plot(p)
dev.off()
```


```
null device 
          1
```

##### 2.2 Financial history - MWRD


```
# Water and Sewer Funds summary
dwm <- read.csv("Money_Data_DWM.csv", header = TRUE, stringsAsFactors = FALSE, sep = ",")
# inflation correction
yr = 2016
i <- which(dwm$year == yr)
dwm <- dwm %>% 
  mutate(infl_corr = CPI_Chicago/CPI_Chicago[i]) 
drops <- names(dwm) %in% c("CPI_Chicago", "infl_corr", "net_position")
dwm[,2:10] <- dwm[,2:10]/dwm$infl_corr
A <- dwm[!drops]
```

##### 2.3 Plotting financial data (manuscript Fig. 4)


```
# --------- MWRD ---------------
mwrd <- read.csv("Money_Data_MWRD.csv", header = TRUE, stringsAsFactors = FALSE, sep = ",")
# inflation correction
yr = 2016
i <- which(mwrd$year == yr)
mwrd <- mwrd %>% 
  mutate(infl_corr = CPI_Chicago/CPI_Chicago[i]) 
drops <- names(mwrd) %in% c("CPI_Chicago", "infl_corr", "net.position.EOY")
mwrd[,2:7] <- mwrd[,2:7]/mwrd$infl_corr
names(mwrd)[names(mwrd) == 'net.position.EOY'] <- 'net_pos'
B <- mwrd[!drops]
```

LS0tDQp0aXRsZTogJycNCm91dHB1dDoNCiAgaHRtbF9kb2N1bWVudDoNCiAgICBjb2RlX2ZvbGRpbmc6IGhpZGUNCiAgaHRtbF9ub3RlYm9vazogZGVmYXVsdA0KICBwZGZfZG9jdW1lbnQ6IGRlZmF1bHQNCiAgd29yZF9kb2N1bWVudDogZGVmYXVsdA0KLS0tDQoNCmBgYHtyIHNldHVwLCBpbmNsdWRlPUZBTFNFfQ0Ka25pdHI6Om9wdHNfY2h1bmskc2V0KHdhcm5pbmcgPSBGQUxTRSwgbWVzc2FnZT1GQUxTRSwgZWNobz1UUlVFKQ0KYGBgDQoNCmBgYHtyLCBpbmNsdWRlfQ0KbGlicmFyeShDaXR5V2F0ZXJCYWxhbmNlKQ0KbGlicmFyeShkYXRhUmV0cmlldmFsKQ0KbGlicmFyeShsZWFmbGV0KQ0KbGlicmFyeShnZ3Bsb3QyKQ0KbGlicmFyeSh0aWR5dmVyc2UpDQpsaWJyYXJ5KHh0cykNCmxpYnJhcnkoZ3JpZEV4dHJhKQ0KbGlicmFyeShrbml0cikNCmBgYA0KDQojIyMgU3VwcGxlbWVudGFyeSBtYXRlcmlhbCBmb3IgIipCZXlvbmQgb2xkIHBpcGVzIGFuZCBhaWxpbmcgYnVkZ2V0czogc3lzdGVtcyB0aGlua2luZyBvbiAyMXN0IGNlbnR1cnkgd2F0ZXIgaW5mcmFzdHJ1Y3R1cmUgaW4gQ2hpY2FnbyoiDQoNCiMjIyMgQXV0aG9yczogTGF1cmEgRSBFcmJhbiBhbmQgSGVucnkgQSBXYWxrZXINCg0KVGhpcyBub3RlYm9vayBwcm92aWRlcyB0aGUgc3RlcHMgdXNlZCB0byBnYXRoZXIsIHN1bW1hcml6ZSBhbmQgcGxvdCB0aGUgd2F0ZXIgYW5kIGZpbmFuY2lhbCBkYXRhIHNob3duIGluIHRoZSBwYXBlciAoRmlncyAzLTQpLiBXaXRoIHRoZSBzdXBwbGVtZW50YXJ5IGRhdGEgZmlsZXMgYW5kIHRoZSBjb2RlIGdpdmVuIGJlbG93LCBvbmUgY2FuIHJlcHJvZHVjZSBvdXIgd29yay4gIA0KDQoNCg0KIyMjIyAxLiBXYXRlciANCg0KIyMjIyMgMS4xIEVudmlyb25tZW50YWwgZmxvd3Mgb2Ygd2F0ZXINCg0KVGhlIGNvZGUgY2h1bmsgdGhhdCBmb2xsb3dzIHdhcyB1c2VkIHRvIGFjcXVpcmUgbW9udGhseSBlc3RpbWF0ZXMgb2YgcHJlY2lwaXRhdGlvbiAocHJjcCkgYW5kIGV2YXBvdHJhbnNwaXJhdGlvbiAoZXQpIHJhdGVzIGZyb20gZ3JpZGRlZCBtb2RlbCBvdXRwdXQgKG1vZGVscyBhcmUgUFJJU00sIFNTRUJvcCwgcmVzcGVjdGl2ZWx5KS4gTW9kZWwgb3V0cHV0IHdhcyByZXRyaWV2ZWQgZnJvbSB0aGUgW1VTR1MncyBHZW8gRGF0YSBQb3J0YWxdKGh0dHBzOi8vY2lkYS51c2dzLmdvdi9nZHAvKSB0aHJvdWdoIHRoZSBlbmQgb2YgMjAxNSBhbmQgc3BhdGlhbGx5IGF2ZXJhZ2VkIHdpdGhpbiB0aGUgYm91bmRhcmllcyBvZiBDb29rIENvdW50eSAoY291bnR5IGJvdW5kYXJpZXMgYXJlIGRhc2hlZCBsaW5lcyBpbiB0aGUgbWFwIGJlbG93LCB3aGljaCBpcyBjZW50ZXJlZCBvbiBDb29rKS4gIFdlIGNhbGN1bGF0ZWQgYW5udWFsIHRvdGFscyAoYXMgZGVwdGggb2Ygd2F0ZXIpIHRocm91Z2ggMjAxNSBhbmQgbWFudWFsbHkgYWRkZWQgcHJlY2lwaXRhdGlvbiB0b3RhbHMgZm9yIDIwMTYgYW5kIDIwMTcgdXNpbmcgZGF0YSBmb3IgTydIYXJlIGFpcnBvcnQgZnJvbSB3ZWF0aGVyLmdvdi4gIFdlIGNvbnZlcnRlZCBkZXB0aHMgdG8gdm9sdW1lcyBieSBtdWx0aXBseWluZyBieSB0aGUgbGFuZCBhcmVhIG9mIENvb2sgQ291bnR5IChpLmUuLCBleGNsdWRpbmcgQ29vaydzIGFyZWEgaW4gTGFrZSBNaWNoaWdhbikuIFdlIHJldHJpZXZlZCBkYWlseSBzdHJlYW1mbG93IGZyb20gW1VTR1MncyBOV0lTXShodHRwczovL3dhdGVyZGF0YS51c2dzLmdvdi9ud2lzKSBmb3Igc3RyZWFtZ2F1Z2VzIG5lYXIgQ29vaydzIGJvdW5kYXJpZXMgKHNlZSBtYXApLiAgU3RyZWFtZmxvdyByZWNvcmRzIGFyZSBub3QgaWRlYWxseSBsb2NhdGVkLCBhbmQgY29udGFpbiBnYXBzLiAgVGhlIGNvZGUgc3BlY2lmaWVzIGhvdyB3ZSBhY2NvdW50ZWQgZm9yIHRob3NlIGlzc3VlcyBhbmQgc3VtbWFyaXplZCB0b3RhbCBhbm51YWwgc3RyZWFtZmxvdyBpbnRvIENvb2sgQ291bnR5Lg0KDQpgYGB7ciwgZXZhbCA9IEZBTFNFfQ0KDQojIGdldCBwcmVjaXBpdGF0aW9uIChwcmNwKQ0KcHJjcCA8LSBnZXRQcmVjaXBpdGF0aW9uKCcxOTk1LTAxLTAxJywgJzIwMTUtMTItMDEnLCAnc2FtcGxlOkNvdW50aWVzJywgJ0ZJUFMnLCcxNzAzMScpDQpuYW1lcyhwcmNwKSA8LSBjKCdkYXRlJywgJ3JhdGUnLCAndmFyJywgJ3N0YXQnLCAndW5pdHMnKQ0KcHN1bSA8LSBwcmNwICU+JSANCiAgbXV0YXRlKHllYXIgPSBsdWJyaWRhdGU6OnllYXIoZGF0ZSkpICU+JQ0KICBncm91cF9ieSh5ZWFyKSAlPiUNCiAgc3VtbWFyaXNlKHB0b3QgPSBzdW0ocmF0ZSkpDQoNCiMgYWRkIGRhdGEgZnJvbSB3ZWF0aGVyLmdvdiBmb3IgTydIYXJlIA0KcHN1bSA8LSByYmluZChwc3VtLCBjKDIwMTYsIDM1Ljk3KjI1LjQpLCBjKDIwMTcsIDQzLjEqMjUuNCkpDQoNCiMgZ2V0IGV2YXBvdHJhbnNwaXJhdGlvbiAoZXQpIA0KZXQgPC0gZ2V0RXZhcG90cmFuc3BpcmF0aW9uKCcxOTk1LTAxLTAxJywgJzIwMTUtMTItMDEnLCAnc2FtcGxlOkNvdW50aWVzJywgJ0ZJUFMnLCcxNzAzMScpIA0KbmFtZXMoZXQpIDwtIGMoJ2RhdGUnLCAncmF0ZScsICd2YXInLCAnc3RhdCcsICd1bml0cycpDQplc3VtIDwtIGV0ICU+JSANCiAgbXV0YXRlKHllYXIgPSBsdWJyaWRhdGU6OnllYXIoZGF0ZSkpICU+JQ0KICBncm91cF9ieSh5ZWFyKSAlPiUNCiAgc3VtbWFyaXNlKGV0b3QgPSBzdW0ocmF0ZSkpDQoNCmF0bSA8LSBsZWZ0X2pvaW4ocHN1bSwgZXN1bSkNCg0KIyBjb252ZXJ0IHByY3AsIGV0IHJhdGVzIHRvIHZvbHVtZXMNCmFyZWEgPC0gMjQ1MCAgICAgIyBsYW5kIGFyZWEgb2YgY29vayBjb3VudHkgKGttMikNCmNmIDwtIDAuMDAzNzg1ICAgIyBjdWJpYyBrbSBwZXIgYmlsbGlvbiBnYWxsb25zICANCmF0bXZvbCA8LSBhdG0gJT4lDQogIG11dGF0ZShwdG90ID0gKHB0b3QvMWU2KSphcmVhL2NmKSAlPiUNCiAgbXV0YXRlKGV0b3QgPSAoZXRvdC8xZTYpKmFyZWEvY2YpDQoNCiMgZ2V0IHN0cmVhbWZsb3cgYW5kIGVzdGltYXRlIHZhbHVlcyBpbiBkYXRhIGdhcHMNCnN0cmVhbWdhZ2VzIDwtIGMoJzA1NTI4MTAwJywnMDU1MjkwMDAnLCcwNTUzNjE5NScsJzA1NTM1MDcwJywgJzA1NTM0NTAwJywgJzA1NTM1NTAwJykNCnMgPC0gZ2V0U3RyZWFtZmxvdygnMTk5NS0wMS0wMScsICcyMDE3LTEyLTMxJywgc3RyZWFtZ2FnZXMpDQpzMiA8LSBnYXBmaWxsU3RyZWFtZmxvdyhzLCBsaXN0KGMoJzA1NTI5MDAwJywnMDU1MjgxMDAnKSwgYygnMDU1MzYxOTUnLCcwNTUyOTAwMCcpLCBjKCcwNTUzNTA3MCcsJzA1NTM2MTk1JyksIGMoJzA1NTM0NTAwJywnMDU1MzYxOTUnKSwgYygnMDU1MzU1MDAnLCcwNTUzNjE5NScpKSkNCnNmIDwtIGFwcGx5LnllYXJseSgoczIkZmxvd3MgKiA2MCo2MCoyNCksIEZVTiA9IGNvbFN1bXMpICAgIyBjb252ZXJ0IHRvIGN1YmljIGZ0IC8gZGF5IGFuZCBzdW1hcml6ZSBieSB5ZWFyDQpzZiA8LSBzZiAqIDcuNDgwNTIgLyAxZTkgICAgICAgICAgICAgICAgICAgICAgICAgICAgICAgICAgICMgY29udmVydCBzdHJlYW1mbG93IHRvIGJpbGxpb24gZ2FsbCAvIHllYXINCg0KIyBkZWFsIHdpdGggc3RyZWFtZ2FnZSBsb2NhdGlvbiBpc3N1ZXMuIGZpcnN0IHR3byBnYWdlcyBhcmUgb24gZGVzIHBsYWluZXMsIH4gZXF1aWRpc3RhbnQgb24gZWl0aGVyIHNpZGUgb2YgQ29vayBDb3VudHkgYm9yZGVyLiAgVXBzdHJlYW0gYXZncyA3MCUgb2YgZG93bnN0cmVhbS4gIEFwcGx5aW5nIDg1JSB0byBkb3duc3RyZWFtIGdhZ2UgKGNvbXBsZXRlIHJlY29yZCkgdG8gc3BsaXQgdGhlIGRpZmZlcmVuY2UNCnNlc3QgPC0gZGF0YS5mcmFtZShsdWJyaWRhdGU6OnllYXIoaW5kZXgoc2YpKSwgYXMudmVjdG9yKHNmWywyXSowLjg1K3NmWywzXStzZlssNF0rc2ZbLDVdK3NmWyw2XSkpDQpuYW1lcyhzZXN0KSA8LSBjKCd5ZWFyJywgJ3N0cmVhbWZsb3dfaW4nKQ0KDQplbnYgPC0gbGVmdF9qb2luKHNlc3QsIGF0bXZvbCkNCmBgYA0KDQojIyMjIyAxLjEuMiBNYXAgc2hvd2luZyBjb3VudHkgYm91bmRhcmllcywgcml2ZXJzIGFuZCBzdHJlYW1nYWdlIGxvY2F0aW9ucw0KDQpgYGB7cn0NCg0Kc3RyZWFtZ2FnZXMgPC0gYygnMDU1MjgxMDAnLCcwNTUyOTAwMCcsJzA1NTM2MTk1JywnMDU1MzUwNzAnLCAnMDU1MzQ1MDAnLCAnMDU1MzU1MDAnKQ0Kc2cgPC0gcmVhZE5XSVNzaXRlKHN0cmVhbWdhZ2VzKQ0KDQojIGxhdCBsb25zIGZvciBzdHJlYW1nYXVnZXMNCnNnbG9jcyA8LSB0aWJibGUobGF0ID0gc2ckZGVjX2xhdF92YSwgbG9uID0gc2ckZGVjX2xvbmdfdmEsIHNpdGUgPSBzZyRzdGF0aW9uX25tKQ0KDQpsZWFmbGV0KHNnbG9jcykgJT4lIA0KICBhZGRQcm92aWRlclRpbGVzKCJFc3JpLldvcmxkVG9wb01hcCIpICU+JQ0KICBhZGRNYXJrZXJzKH5sb24sIH5sYXQsIHBvcHVwID0gfmFzLmNoYXJhY3RlcihzaXRlKSwgbGFiZWwgPSB+YXMuY2hhcmFjdGVyKHNpdGUpKSAlPiUNCiAgYWRkU2NhbGVCYXIoKQ0KYGBgDQoNCg0KIyMjIyMgMS4yIE1hbm1hZGUgZmxvd3MNCg0KVGhlIGNvZGUgY2h1bmsgYmVsb3cgbG9hZHMgdGhlIGRhdGEgZ2F0aGVyZWQgYWJvdmUgYW5kIGFkZGl0aW9uYWwgZGF0YSBvbiBtYW5tYWRlIGZsb3dzIG9mIHdhdGVyLCBtb3N0IHNvdXJjZXMgb2Ygd2hpY2ggYXJlIG5vdCBmZWRlcmF0ZWQuICBXYXRlciB1c2UgZGF0YSBhdCB0aGUgY291bnR5IGxldmVsIGFyZSBwdWJsaXNoZWQgYnkgVVNHUyBhdCA1LXllYXIgaW50ZXJ2YWxzIGZyb20gMTk4NS0yMDE1LiBDb2RlIGJlbG93IHNob3dzIGhvdyB0byByZXRyaWV2ZSB0aGVzZSBkYXRhLiAgV2FzdGV3YXRlciBlZmZsdWVudCAoaS5lLiB0cmVhdGVkIHdhc3Rld2F0ZXIpIGRhdGEgd2FzIG1hbnVhbGx5IGRvd25sb2FkZWQgZnJvbSBbTVdSRCdzIFdhdGVyIFJlY2xhbWF0aW9uIFBsYW50IERhdGFdKGh0dHA6Ly93d3cubXdyZC5vcmcvaXJqL3BvcnRhbC9hbm9ueW1vdXM/TmF2aWdhdGlvblRhcmdldD1uYXZ1cmw6Ly85Zjc2NmQ0ZjgyMGU5NDgyZDAxNjY4MWM4NjAzMWI3NiksIGF2YWlsYWJsZSBhcyBFeGNlbCBzcHJlYWRzaGVldHMgYnkgZGF5IGFuZCBwbGFudCBnb2luZyBiYWNrIHRvIDE5ODIuIENvbWJpbmVkIHNld2VyIG92ZXJmbG93IChDU08pIHZvbHVtZXMgd2VyZSBvYnRhaW5lZCB0aHJvdWdoIGEgd3JpdHRlbiByZXF1ZXN0IHRvIE1XUkQuIFB1bXBhZ2UgYnkgRFdNIGZyb20gTGFrZSBNaWNoaWdhbiBpcyBkaXNjbG9zZWQgaW4gQ0FGUnMgKGRpc2N1c3NlZCBtb3JlIGluIHRoZSBmaW5hbmNpYWxzIHNlY3Rpb24gYmVsb3cpLiBXZSBhZ2dyZWdhdGVkIHRoZXNlIGRhdGEgb3V0c2lkZSBvZiB0aGlzIHdvcmtib29rIGFuZCBwcm92ZSBhbm51YWwgc3VtbWFyaWVzLCBhbG9uZyB3aXRoIHRoZSBlbnZpcm9ubWVudGFsIGZsb3cgZGF0YSBmcm9tIGFib3ZlLCBpbiB0aGUgc3VwcGxlbWVudGFyeSBmaWxlICJXYXRlcl9EYXRhLmNzdiIuDQoNCmBgYHtyLCBldmFsID0gRkFMU0V9DQoNCiMgLS0tLS0tLSByZXRyaWV2ZSBwdWJsaWMgc3VwcGx5IHdpdGhkcmF3YWxzIC0tLS0tLS0NCnd1IDwtIGdldFdhdGVyVXNlKGMoJ0lMJyksIGxpc3QoYygnQ29vaycpKSkNCg0KIyBhZ2dyZWdhdGUgc3VyZmFjZSwgZ3JvdW5kd2F0ZXIuICBjb3ZlcnQgdG8gQkcgLyB5cg0KcHVidyA8LSAod3Ukc3dmJFB1YmxpYyArIHd1JGd3ZiRQdWJsaWMpICogMzY1LzEwMDANCnB1YncgPC0gZGF0YS5mcmFtZSh3dSRzd2YkeWVhciwgcHVidykNCm5hbWVzKHB1YncpIDwtIGMoJ3llYXInLCAncHVibGljX3N1cHBseV9CRycpDQojIC0tLS0tLS0tLS0tLS0tLS0tLS0tLS0tLS0tLS0tLS0tLS0tLS0tLS0tLS0tLS0tLS0tDQoNCiMgbG9hZCBhbGwgd2F0ZXIgZGF0YQ0Kd2F0ZXIgPC0gcmVhZC5jc3YoIldhdGVyX0RhdGEuY3N2IikNCmBgYA0KDQojIyMjIyAxLjMgV2F0ZXIgZmxvdyBkYXRhIHBsb3R0ZWQgKG1hbnVzY3JpcHQgRmlnLiAzKQ0KYGBge3IsIGZpZy5oZWlnaHQgPSA0LCBmaWcud2lkdGggPSA4fQ0KDQojIGxvYWQgYWxsIHdhdGVyIGRhdGENCndhdGVyIDwtIHJlYWQuY3N2KCJXYXRlcl9EYXRhLmNzdiIpDQoNCiMgMS4gUGxvdCBidWxrIHdhdGVyIGZsb3dzDQptIDwtIGdhdGhlcih3YXRlciwgdmFyLCB2YWx1ZSwgLXllYXIpDQptJHZhciA8LSBmYWN0b3IobSR2YXIsIGxldmVscyA9IHVuaXF1ZShtJHZhcikpDQoNCnNoYXBlcyA9IGMoMTYsIDE1LCAxNywgMTksIE5BLCA0LCAxKQ0KDQpwIDwtIGdncGxvdChtLCBhZXMoeCA9IHllYXIsIHkgPSB2YWx1ZSwgZ3JvdXAgPSB2YXIpKSArDQogIGdlb21fbGluZShhZXMoY29sb3VyID0gdmFyKSwgc2l6ZSA9IDEpICsNCiAgZ2VvbV9wb2ludChhZXMoY29sb3VyID0gdmFyLCBzaGFwZSA9IHZhciksIHNpemUgPSAyKSArDQogIHNjYWxlX3NoYXBlX21hbnVhbCh2YWx1ZXMgPSBzaGFwZXMsIGd1aWRlID0gJ25vbmUnKSArDQogIHNjYWxlX3hfY29udGludW91cyhsaW1pdHMgPSBjKDE5OTUsIDIwMTcpLCBicmVha3MgPSBzZXEoMTk5MCwgMjAxNywgNSksIGV4cGFuZCA9IGMoMC4wMSwgMC4wMSkpICsNCiAgc2NhbGVfeV9jb250aW51b3VzKGxpbWl0cyA9IGMoMCwgMTAwMCksIGV4cGFuZCA9IGMoMC4wMiwgMC4wMikpICsgDQogIHRoZW1lX2J3KCkgKw0KICBzY2FsZV9jb2xvdXJfbWFudWFsKG5hbWUgPSAnJywgdmFsdWVzID0gYygnYmx1ZScsICdzZWFncmVlbjMnLCAnY29ybmZsb3dlcmJsdWUnLCAnZGFya29yY2hpZCcsICdkYXJrb3JjaGlkMicsICdkYXJrb3JhbmdlJywgJ2RhcmtvcmFuZ2U0JyksIGxhYmVscyA9IGMoJ1ByZWNpcGl0YXRpb24nLCAnRXZhcG90cmFuc3BpcmF0aW9uJywgJ1N0cmVhbWZsb3cgaW4nLCAnQ29vayBDb3VudHkgd2l0aGRyYXdhbHMnLCAnRFdNICB3aXRoZHJhd2FscycsICdNV1JEIGVmZmx1ZW50JywgJ0NvbWJpbmVkIHNld2VyIG92ZXJmbG93JykpICsNCiAgZ2d0aXRsZShwYXN0ZSgiV2F0ZXIgZmxvd3MiKSkgKw0KICBsYWJzKHggPSAiIiwgeSA9ICJBbm51YWwgXG4gdm9sdW1lIFxuIFxuIChiaWxsaW9uIFxuIGdhbGxvbnMpIikgKw0KICBndWlkZXMoY29sb3VyID0gZ3VpZGVfbGVnZW5kKG92ZXJyaWRlLmFlcyA9IGxpc3QobGluZXR5cGUgPSBjKDEsIDEsIDEsIDAsIDEsIDEsIDEpLCBzaGFwZSA9IHNoYXBlcykpKSArDQogIHRoZW1lKHBsb3QudGl0bGUgPSBlbGVtZW50X3RleHQoc2l6ZSA9IDEyLCBmYWNlID0gImJvbGQiKSwgIGxlZ2VuZC50ZXh0ID0gZWxlbWVudF90ZXh0KHNpemUgPSAxMCksIGxlZ2VuZC5wb3NpdGlvbiA9ICJyaWdodCIsIGF4aXMudGl0bGUueSA9IGVsZW1lbnRfdGV4dChhbmdsZSA9IDAsIHZqdXN0ID0gMC41KSkNCg0KcGxvdChwKQ0KYGBgDQoNCg0KIyMjIyAyLiAgTW9uZXkNCg0KRmxvd3Mgb2YgbW9uZXkgdGhyb3VnaCB0aGUgdHdvIHdhdGVyIHV0aWxpdGllcyBzZXJ2aW5nIENoaWNhZ28gKERXTSwgTVdSRCkgd2VyZSBleHRyYWN0ZWQgZnJvbSBDb21wcmVoZW5zaXZlIEFubnVhbCBGaW5hbmNpYWwgUmVwb3J0cyAoQ0FGUikuIEZpbmFuY2lhbCBmbG93cyB0aHJvdWdoIERXTSB3ZXJlIHN1bW1lZCBmcm9tIHZhbHVlcyBpbiB0d28gc2V0cyBvZiBiaWVubmlhbCBbQ0FGUnMgZm9yIENoaWNhZ28ncyBXYXRlciBhbmQgU2V3ZXIgRnVuZHNdKGh0dHBzOi8vd3d3LmNpdHlvZmNoaWNhZ28ub3JnL2NpdHkvZW4vZGVwdHMvZmluL3N1cHBfaW5mby9jb21wcmVoZW5zaXZlX2FubnVhbGZpbmFuY2lhbHN0YXRlbWVudHMuaHRtbCksIHB1Ymxpc2hlZCBvbmxpbmUgZm9yIDIwMDYtMjAxNi4gVmFsdWVzIHdlcmUgZXh0cmFjdGVkIGZyb20gYSBzaW5nbGUgc2V0IG9mIFtNV1JEIENBRlJzXShodHRwczovL3d3dy5td3JkLm9yZy9pcmovc2VydmxldC9wcnQvcG9ydGFsL3BydHJvb3QvcGNkITNhcG9ydGFsX2NvbnRlbnQhMmZNV1JEITJmTVdSREludGVybmV0ITJmUm9sZXMhMmZSZXBvcnRzITJmRmluYW5jaWFsITJmQW5udWFsX1JlcG9ydCEyZkZpbmFuY2VfUmVwb3J0KSwgYXZhaWxhYmxlIG9uIGEgYmllbm5pYWwgYmFzaXMgZnJvbSAyMDA0LTIwMTYuIFRoaXMgd2FzIGFsc28gYSBtYW51YWwgcHJvY2VzcywgcmVzdWx0cyBvZiB3aGljaCBhcmUgcHJvdmlkZWQgaW4gIk1vbmV5X0RhdGFfRFdNLmNzdiIgYW5kICJNb25leV9EYXRhX01XUkQuY3N2Ii4gIEluIHRoZSBjb2RlIGNodW5rIGJlbG93LCB0aGV5IGFyZSBjb3JyZWN0ZWQgZm9yIGluZmxhdGlvbiAocmVsYXRpdmUgdG8gMjAxNiksIHVzaW5nIHRoZSBbQ29uc3VtZXIgUHJpY2UgSW5kZXggZm9yIEFsbCBVcmJhbiBDb25zdW1lcnMgKENQSS1VKSBDaGljYWdvLU5hcGVydmlsbGUtRWxnaW5dKGh0dHBzOi8vd3d3LmJscy5nb3YvcmVnaW9ucy9taWR3ZXN0L25ld3MtcmVsZWFzZS9jb25zdW1lcnByaWNlaW5kZXhfY2hpY2Fnby5odG0pIGF2YWlsYWJsZSBmcm9tIHRoZSBVUyBCdXJlYXUgb2YgTGFib3IgU3RhdGlzdGljcy4gDQoNCiMjIyMjIDIuMSBGaW5hbmNpYWwgaGlzdG9yeSAtIERXTQ0KYGBge3IsIGZpZy5oZWlnaHQgPSA0LCBmaWcud2lkdGggPSA4fSANCg0KIyBXYXRlciBhbmQgU2V3ZXIgRnVuZHMgc3VtbWFyeQ0KZHdtIDwtIHJlYWQuY3N2KCJNb25leV9EYXRhX0RXTS5jc3YiLCBoZWFkZXIgPSBUUlVFLCBzdHJpbmdzQXNGYWN0b3JzID0gRkFMU0UsIHNlcCA9ICIsIikNCg0KIyBpbmZsYXRpb24gY29ycmVjdGlvbg0KeXIgPSAyMDE2DQppIDwtIHdoaWNoKGR3bSR5ZWFyID09IHlyKQ0KZHdtIDwtIGR3bSAlPiUgDQogIG11dGF0ZShpbmZsX2NvcnIgPSBDUElfQ2hpY2Fnby9DUElfQ2hpY2Fnb1tpXSkgDQoNCmRyb3BzIDwtIG5hbWVzKGR3bSkgJWluJSBjKCJDUElfQ2hpY2FnbyIsICJpbmZsX2NvcnIiLCAibmV0X3Bvc2l0aW9uIikNCg0KZHdtWywyOjEwXSA8LSBkd21bLDI6MTBdL2R3bSRpbmZsX2NvcnINCkEgPC0gZHdtWyFkcm9wc10NCmBgYA0KDQojIyMjIyAyLjIgRmluYW5jaWFsIGhpc3RvcnkgLSBNV1JEIA0KYGBge3J9DQoNCiMgLS0tLS0tLS0tIE1XUkQgLS0tLS0tLS0tLS0tLS0tDQptd3JkIDwtIHJlYWQuY3N2KCJNb25leV9EYXRhX01XUkQuY3N2IiwgaGVhZGVyID0gVFJVRSwgc3RyaW5nc0FzRmFjdG9ycyA9IEZBTFNFLCBzZXAgPSAiLCIpDQoNCiMgaW5mbGF0aW9uIGNvcnJlY3Rpb24NCnlyID0gMjAxNg0KaSA8LSB3aGljaChtd3JkJHllYXIgPT0geXIpDQptd3JkIDwtIG13cmQgJT4lIA0KICBtdXRhdGUoaW5mbF9jb3JyID0gQ1BJX0NoaWNhZ28vQ1BJX0NoaWNhZ29baV0pIA0KDQpkcm9wcyA8LSBuYW1lcyhtd3JkKSAlaW4lIGMoIkNQSV9DaGljYWdvIiwgImluZmxfY29yciIsICJuZXQucG9zaXRpb24uRU9ZIikNCg0KbXdyZFssMjo3XSA8LSBtd3JkWywyOjddL213cmQkaW5mbF9jb3JyDQpuYW1lcyhtd3JkKVtuYW1lcyhtd3JkKSA9PSAnbmV0LnBvc2l0aW9uLkVPWSddIDwtICduZXRfcG9zJw0KQiA8LSBtd3JkWyFkcm9wc10NCmBgYA0KDQojIyMjIyAyLjMgUGxvdHRpbmcgZmluYW5jaWFsIGRhdGEgKG1hbnVzY3JpcHQgRmlnLiA0KQ0KDQpgYGB7ciwgZmlnLmhlaWdodCA9IDksIGZpZy53aWR0aCA9IDh9DQoNCiMgLS0tLS0tLS0tLS0tLS0gRFdNIC0tLS0tLS0tLS0tLS0tLS0tLS0tLQ0KbSA8LSBnYXRoZXIoQSwgdmFyLCB2YWx1ZSwgLXllYXIpDQptJHZhciA8LSBmYWN0b3IobSR2YXIsIGxldmVscyA9IHVuaXF1ZShtJHZhcikpDQoNCnNoYXBlczEgPSBjKDE2LCAxNSwgMCwgMSwgMiwgNSwgNiwgNykNCg0KcDEgPC0gZ2dwbG90KG0sIGFlcyh4ID0geWVhciwgeSA9IHZhbHVlLCBncm91cCA9IHZhcikpICsNCiAgZ2VvbV9saW5lKGFlcyhjb2xvdXIgPSB2YXIpLCBzaXplID0gMSkgKw0KICBnZW9tX3BvaW50KGFlcyhjb2xvdXIgPSB2YXIsIHNoYXBlID0gdmFyKSwgc2l6ZSA9IDIpICsNCiAgc2NhbGVfc2hhcGVfbWFudWFsKHZhbHVlcyA9IHNoYXBlczEsIGd1aWRlID0gJ25vbmUnKSArDQogIHNjYWxlX3hfY29udGludW91cyhsaW1pdHMgPSBjKDE5OTUsIDIwMTcpLCBicmVha3MgPSBzZXEoMTk5MCwgMjAxNywgNSksIGV4cGFuZCA9IGMoMC4wMSwgMC4wMSkpICsNCiAgc2NhbGVfeV9jb250aW51b3VzKGxpbWl0cyA9IGMoMCwgMTIwMCksIGV4cGFuZCA9IGMoMC4wMiwgMC4wMikpICsgDQogIHRoZW1lX2J3KCkgKw0KICBzY2FsZV9jb2xvdXJfbWFudWFsKG5hbWUgPSAnJywgdmFsdWVzID0gYygnbWVkaXVtc2VhZ3JlZW4nLCAnZGFya2dyZWVuJywgJ2RhcmtvcmFuZ2UnLCAnY3lhbjMnLCAnZGFya29yY2hpZDInLCAndmlvbGV0cmVkMicsICdkYXJrZ29sZGVucm9kMicsICdyb3lhbGJsdWUnKSwgbGFiZWxzID0gYygnVG90YWwgcmV2ZW51ZXMnLCAnICAgIFNhbGVzJywgJ09wZXJhdGluZyBleHBlbnNlcycsICcgICAgV2F0ZXIgYW5kIHNld2VyJywgJyAgICBQZW5zaW9uIGNvbnRyaWJ1dGlvbnMnLCAnICAgIFBlbnNpb24gZXhwZW5zZScsICcgICAgT3RoZXIgY2l0eSBkZXBhcnRtZW50cycsICdOb24tb3BlcmF0aW5nIGV4cGVuc2VzJykpICsNCiAgZ2d0aXRsZShwYXN0ZSgiQS4gIEZpbmFuY2lhbCBmbG93czogRFdNICAoMjAxNiBkb2xsYXJzKSIpKSArDQogIGxhYnMoeCA9ICIiLCB5ID0gIkFubnVhbCBcbiBhbW91bnQgXG4gXG4gKG1pbGxpb24gXG4gZG9sbGFycykiKSArDQogIGd1aWRlcyhjb2xvdXIgPSBndWlkZV9sZWdlbmQob3ZlcnJpZGUuYWVzID0gbGlzdChsaW5ldHlwZSA9IGMoMSwgMSwgMSwgMSwgMSwgMSwgMSwgMSksIHNoYXBlID0gc2hhcGVzMSkpKSArDQogIHRoZW1lKHBsb3QudGl0bGUgPSBlbGVtZW50X3RleHQoc2l6ZSA9IDEyLCBmYWNlID0gImJvbGQiKSwgIGxlZ2VuZC50ZXh0ID0gZWxlbWVudF90ZXh0KHNpemUgPSAxMCksIGxlZ2VuZC5wb3NpdGlvbiA9ICJyaWdodCIsIGF4aXMudGl0bGUueSA9IGVsZW1lbnRfdGV4dChhbmdsZSA9IDAsIHZqdXN0ID0gMC41KSkgDQoNCiMgLS0tLS0tLS0tLS0tLS0gTVdSRCAtLS0tLS0tLS0tLS0tLS0tLS0tLQ0KbTIgPC0gZ2F0aGVyKEIsIHZhciwgdmFsdWUsIC15ZWFyKQ0KbTIkdmFyIDwtIGZhY3RvcihtMiR2YXIsIGxldmVscyA9IHVuaXF1ZShtMiR2YXIpKQ0KDQpzaGFwZXMyID0gYygxNiwgMTUsIDAsIDEsIDIpDQoNCnAyIDwtIGdncGxvdChtMiwgYWVzKHggPSB5ZWFyLCB5ID0gdmFsdWUsIGdyb3VwID0gdmFyKSkgKw0KICBnZW9tX2xpbmUoYWVzKGNvbG91ciA9IHZhciksIHNpemUgPSAxKSArDQogIGdlb21fcG9pbnQoYWVzKGNvbG91ciA9IHZhciwgc2hhcGUgPSB2YXIpLCBzaXplID0gMikgKw0KICBzY2FsZV9zaGFwZV9tYW51YWwodmFsdWVzID0gc2hhcGVzMiwgZ3VpZGUgPSAnbm9uZScpICsNCiAgc2NhbGVfeF9jb250aW51b3VzKGxpbWl0cyA9IGMoMTk5NSwgMjAxNyksIGJyZWFrcyA9IHNlcSgxOTkwLCAyMDE3LCA1KSwgZXhwYW5kID0gYygwLjAxLCAwLjAxKSkgKw0KICBzY2FsZV95X2NvbnRpbnVvdXMobGltaXRzID0gYygwLCAxMjAwKSwgZXhwYW5kID0gYygwLjAyLCAwLjAyKSkgKyANCiAgdGhlbWVfYncoKSArDQogIHNjYWxlX2NvbG91cl9tYW51YWwobmFtZSA9ICcnLCB2YWx1ZXMgPSBjKCdtZWRpdW1zZWFncmVlbicsICdkYXJrZ3JlZW4nLCAnZGFya29yYW5nZScsICdkYXJrb3JjaGlkMicsICdyb3lhbGJsdWUnKSwgbGFiZWxzID0gYygnVG90YWwgcmV2ZW51ZXMnLCAnICAgIFRheGVzJywgJ1RvdGFsIGV4cGVuc2VzJywgJyAgICBQZW5zaW9uIGNvc3RzICAgICAgICAgICAnLCAnICAgIEludGVyZXN0IGV4cGVuc2UgICAgICAgICAnKSkgKw0KICBnZ3RpdGxlKHBhc3RlKCJCLiAgRmluYW5jaWFsIGZsb3dzOiBNV1JEICgyMDE2IGRvbGxhcnMpIikpICsNCiAgbGFicyh4ID0gIiIsIHkgPSAiQW5udWFsIFxuIGFtb3VudCBcbiBcbiAobWlsbGlvbiBcbiBkb2xsYXJzKSIpICsNCiAgZ3VpZGVzKGNvbG91ciA9IGd1aWRlX2xlZ2VuZChvdmVycmlkZS5hZXMgPSBsaXN0KGxpbmV0eXBlID0gYygxLCAxLCAxLCAxLCAxKSwgc2hhcGUgPSBzaGFwZXMyKSkpICsNCiAgdGhlbWUocGxvdC50aXRsZSA9IGVsZW1lbnRfdGV4dChzaXplID0gMTIsIGZhY2UgPSAiYm9sZCIpLCAgbGVnZW5kLnRleHQgPSBlbGVtZW50X3RleHQoc2l6ZSA9IDEwKSwgbGVnZW5kLnBvc2l0aW9uID0gInJpZ2h0IiwgYXhpcy50aXRsZS55ID0gZWxlbWVudF90ZXh0KGFuZ2xlID0gMCwgdmp1c3QgPSAwLjUpKQ0KDQojIC0tLS0tLS0tLS0tLS0tIE5ldCBwb3NpdGlvbiwgYm90aCBhZ2VuY2llcw0KDQpib3RoIDwtIGxlZnRfam9pbihkd21bLGMoInllYXIiLCAibmV0X3Bvc2l0aW9uIildLCBtd3JkWyxjKCJ5ZWFyIiwgIm5ldF9wb3MiKV0pDQpuYW1lcyhib3RoKSA8LSBjKCJ5ZWFyIiwgImR3bV9ucCIsICJtd3JkX25wIikNCg0KbTMgPC0gZ2F0aGVyKGJvdGgsIHZhciwgdmFsdWUsIC15ZWFyKQ0KbTMkdmFyIDwtIGZhY3RvcihtMyR2YXIsIGxldmVscyA9IHVuaXF1ZShtMyR2YXIpKQ0KDQpzaGFwZXMzIDwtIGMoMTYsIDE3KQ0KDQpwMyA8LSBnZ3Bsb3QobTMsIGFlcyh4ID0geWVhciwgeSA9IHZhbHVlLCBncm91cCA9IHZhcikpICsNCiAgZ2VvbV9saW5lKGFlcyhjb2xvdXIgPSB2YXIpLCBzaXplID0gMSkgKw0KICBnZW9tX3BvaW50KGFlcyhjb2xvdXIgPSB2YXIsIHNoYXBlID0gdmFyKSwgc2l6ZSA9IDIpICsNCiAgc2NhbGVfc2hhcGVfbWFudWFsKHZhbHVlcyA9IHNoYXBlczMsIGd1aWRlID0gJ25vbmUnKSArDQogIHNjYWxlX3hfY29udGludW91cyhsaW1pdHMgPSBjKDE5OTUsIDIwMTcpLCBicmVha3MgPSBzZXEoMTk5MCwgMjAxNywgNSksIGV4cGFuZCA9IGMoMC4wMSwgMC4wMSkpICsNCiAgc2NhbGVfeV9jb250aW51b3VzKGxpbWl0cyA9IGMoMCwgNjUwMCksIGV4cGFuZCA9IGMoMC4wMiwgMC4wMikpICsgDQogIHRoZW1lX2J3KCkgKw0KICBzY2FsZV9jb2xvdXJfbWFudWFsKG5hbWUgPSAnJywgdmFsdWVzID0gYygnYXF1YW1hcmluZTQnLCAnZGFya29yY2hpZDEnKSwgbGFiZWxzID0gYygnRFdNJywgJ01XUkQgICAgICAgICAgICAgICAgICAgICAgICAgICcpKSArDQogIGdndGl0bGUocGFzdGUoIkMuICBOZXQgcG9zaXRpb24gKDIwMTYgZG9sbGFycykiKSkgKw0KICBndWlkZXMoY29sb3VyID0gZ3VpZGVfbGVnZW5kKG92ZXJyaWRlLmFlcyA9IGxpc3QobGluZXR5cGUgPSBjKDEsIDEpLCBzaGFwZSA9IHNoYXBlczMpKSkgKw0KICBsYWJzKHggPSAiIiwgeSA9ICJBbm51YWwgXG4gYW1vdW50IFxuIFxuIChtaWxsaW9uIFxuIGRvbGxhcnMpIikgKw0KICB0aGVtZShwbG90LnRpdGxlID0gZWxlbWVudF90ZXh0KHNpemUgPSAxMiwgZmFjZSA9ICJib2xkIiksICBsZWdlbmQudGV4dD1lbGVtZW50X3RleHQoc2l6ZSA9IDEwKSwgbGVnZW5kLnBvc2l0aW9uID0gInJpZ2h0IiwgYXhpcy50aXRsZS55ID0gZWxlbWVudF90ZXh0KGFuZ2xlID0gMCwgdmp1c3QgPSAwLjUpKQ0KDQpncmlkLmFycmFuZ2UocDEsIHAyLCBwMywgbnJvdyA9IDMpDQpgYGANCg0KDQo=
